# Study protocol of a randomized controlled trial to assess the efficacy of the “PrEPare for Work” intervention to enhance PrEP uptake and optimize adherence for HIV prevention among male sex workers in the U.S.

**DOI:** 10.1186/s12889-024-17710-y

**Published:** 2024-02-09

**Authors:** Katie B. Biello, Philip A. Chan, Colleen D. Ndoye, Lance Nelson, Elizabeth Nelson, Vanessa Silva, Eun Kwak, Siena Napoleon, Carolina Cormack Orellana, Olly G. Richards, Evan Davis, Matthew J. Mimiaga

**Affiliations:** 1 Department of Behavioral and Social Sciences, Brown University School of Public Health, 121 South Main Street, Providence, RI 02912 USA; 2 Center for Health Promotion and Health Equity Research, Brown University School of Public Health, Providence, USA; 3 The Fenway Institute, Fenway Health, Boston, USA; 4 Department of Medicine, Brown University, Providence, USA; 5 Open Door Health, Rhode Island Public Health Institute, Providence, USA; 6 Project Weber/RENEW, 121 South Main Street, Providence, RI 02912 USA; 7 Department of Epidemiology, UCLA Fielding School of Public Health, Los Angeles, CA USA; 8 Department of Psychiatry & Biobehavioral Sciences, UCLA David Geffen School of Medicine, Los Angeles, CA USA; 9 UCLA Center for LGBTQ+ Advocacy, Research & Health, Los Angeles, CA USA; 10 Department of Epidemiology, Brown University School of Public Health, 121 South Main Street, Providence, RI 02912 USA

**Keywords:** HIV infections, Male sex work, Pre-exposure prophylaxis, Efficacy trial, Motivational interviewing, Social cognitive theory

## Abstract

**Background:**

Male sex workers (MSWs), specifically cisgender men who exchange sex for money, goods, drugs, or other items of value with other cisgender men, are at high risk for HIV infection. Compared to men not engaged in sex work, MSWs are more likely to engage in frequent condomless sex with paying and non-paying sexual partners. While MSWs are often included as a subgroup of gay and bisexual men, data show that a large proportion identify as heterosexual; additionally, most MSWs do not identify as “sex workers.” This places MSWs in a unique position where they may not engage with traditional HIV prevention programs, and when they do, they may not feel comfortable, leading to poor retention. Thus, HIV prevention interventions that address MSWs’ unique life circumstances and provide support in exploring their sexual health options are needed.

**Methods:**

In this protocol paper, we describe the design and procedures for a National Institute of Health-funded, randomized controlled trial testing the efficacy of “PrEPare for Work,”— a theory-based, manualized PrEP uptake and adherence intervention for MSW — using a 2-stage randomization design. Stage 1: MSWs are equally randomized to receive either the “PrEPare for Work Stage 1 intervention” (strength-based case management and facilitated PrEP linkage) or Standard of Care (SOC) to evaluate successful PrEP uptake (prescription filled) within two months post-randomization. Stage 2: Those who initiate PrEP are then equally re-randomized to receive either the “PrEPare for Work Stage 2 intervention” (1-on-1 skills training, problem-solving, and motivational interviewing adherence counseling and personalized, daily text message reminders) or SOC to assess adherence (Tenofovir concentrations in hair) over 12 months of follow up. Planned analyses will examine intervention efficacy, specific conceptual mediators, and hypothesized moderators.

**Discussion:**

Based on our extensive preliminary research, multi-component, theory-informed interventions targeting this subpopulation of MSWs’ unique life circumstances are urgently needed. In this study, we are evaluating whether “PrEPare for Work” can improve PrEP uptake and adherence among MSWs. If this intervention is efficacious, it would be readily disseminated to diverse community organizations that serve MSWs and possibly other community or clinic-based settings.

**Trial registration:**

ClinicalTrials.gov number NCT05736614, registered February 8, 2023.

## Background

Male sex workers (MSWs), specifically cisgender men who exchange anal sex for money, goods, drugs, or other items of value with other cisgender men, are at disproportionately high risk for HIV infection. Our meta-analysis found an estimated HIV prevalence of 20% among cisgender men who have ever engaged in transactional sex in the United States (U.S.), 25 times higher than U.S. men overall [1]. MSWs experience a high burden of structural and psychosocial challenges, such as lack of housing, high unemployment or underemployment, incarceration, substance use, depression, victimization, and discrimination [2–5]. These multi-level challenges are interrelated and associated with sexual behaviors that are associated with increased HIV risk (e.g., condomless anal sex) and reduced access to healthcare and social service—thus, increasing HIV risk [2, 4–7].

Pre-exposure prophylaxis (PrEP) is a once-daily oral pill that is efficacious for the prevention of HIV among uninfected, at-risk individuals [8–10]. Notably, modeling studies estimate that increasing PrEP use among male sex workers specifically would be cost-effective in reducing HIV incidence among MSWs *and* MSM more broadly [11]. However, after over a decade since approval, PrEP uptake in specific at-risk subpopulations of MSWs remains nearly nonexistent [4, 7, 12]. Moreover, many of the challenges that place MSWs at higher risk of HIV also act as barriers to optimal adherence [4, 7, 13]. Thus, any attempt to increase PrEP use among MSWs must be tailored to MSWs’ distinct structural and psychosocial circumstances.

Brief strengths-based case management (SBCM), a participant-driven case management model, is fostered by a peer or professional relationship, wherein the participant identifies and applies their skills and abilities to self-identified, needs-based goal setting and problem-solving. This model has been effective with other high-risk populations, such as substance users [14–16]. Extending the SBCM model to PrEP initiation among MSWs, we hypothesize that SBCM can help individuals identify their strengths and use them to reduce structural, social and personal barriers to PrEP uptake (e.g., transportation, making appointments, health insurance, motivation) with the help of a case manager.

While MSWs face multifaceted challenges to optimal adherence [12, 17, 18], developing and testing an intervention that recognizes and provides strategies to overcome such barriers and implement facilitators to PrEP uptake and adherence among MSWs is necessary to ensure maximum PrEP effectiveness. Efficacious ART adherence interventions for individuals living with HIV can be used as a starting point for addressing these unique challenges. One such intervention, Life-Steps, is an efficacious minimal-treatment behavioral intervention for adherence, which is conducive to adaptation [19–21]. It is based on Social Cognitive Therapy (SCT) and incorporates general principles of Cognitive-Behavioral Therapy (CBT) and Motivational Interviewing (MI) [17, 19–22]. The hypothesized mechanism of action is as follows: the intervention will help MSWs improve motivation, self-regulation, problem-solving skills, and strategies to take medications — all of which are strongly related to adherence and are key constructs of SCT [17, 19–22]. Using a staged intervention approach, we conducted extensive qualitative work to inform the adaptation. We then fielded a pilot RCT of the newly developed intervention to SOC [17]. In addition to demonstrating the feasibility and acceptability of the intervention, we found that participants randomized to “PrEPare for Work” SBCM were two to three times more likely than those in the SOC to attend an initial PrEP appointment, receive a PrEP prescription, and initiate PrEP. Additionally, individuals randomized to receive the “PrEPare for Work” Adherence Counseling intervention were nearly twice as likely to have prevention-effective adherence (measured via hair samples) compared to those in the SOC arm. However, this finding did not reach statistical significance due to insufficient power.

The current paper describes the protocol for a full-scale, multi-site RCT to determine the efficacy of the intervention to increase PrEP initiation and adherence, to examine hypothesized mediators and moderators of the intervention, and, if efficacious, to estimate the cost-effectiveness of the intervention to reduce HIV transmission among MSWs.

## Methods

### Overview of study design

**Fig. 1:**
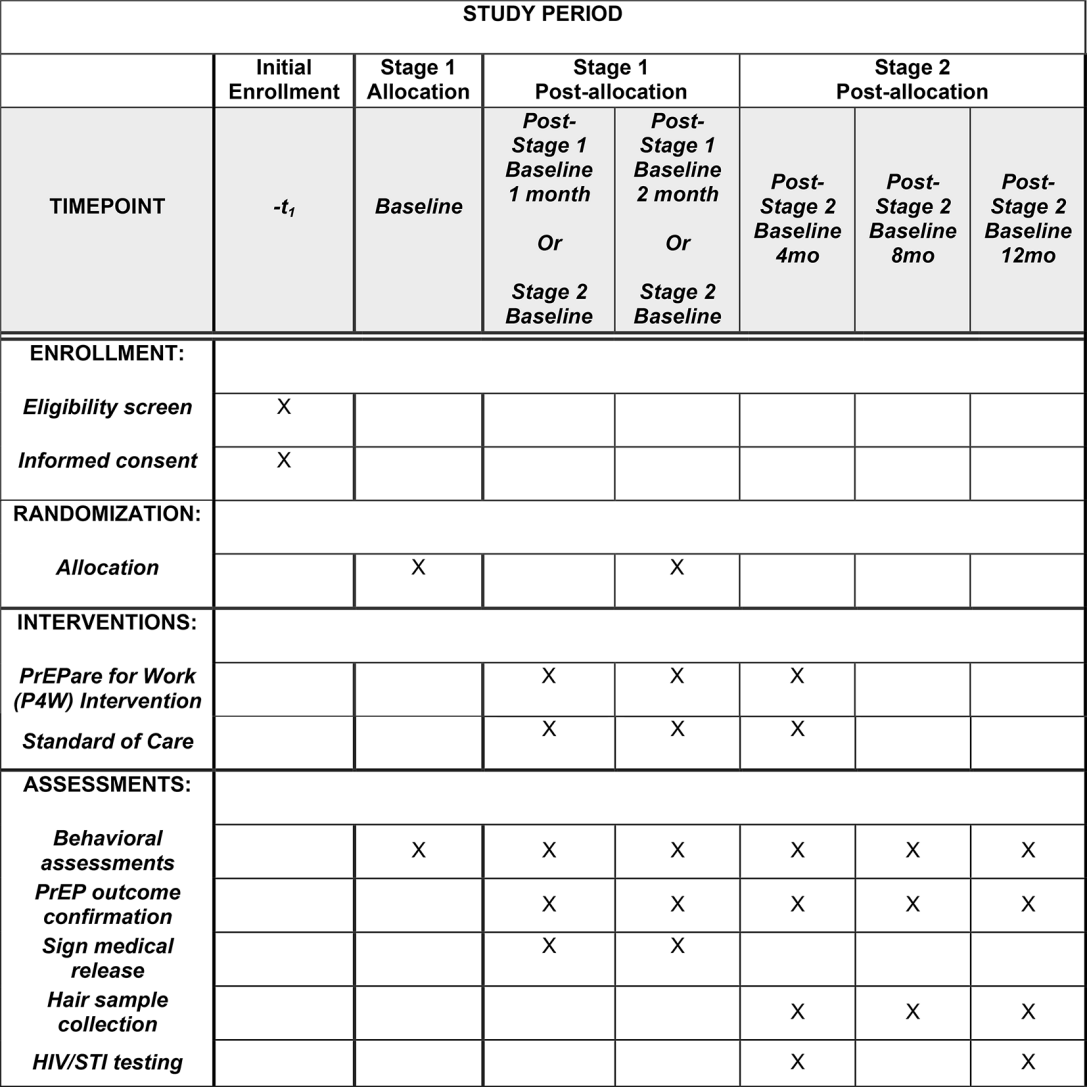
SPIRIT figure for the PrEPare for Work trial

“PrEPare for Work” is a theory-based, manualized intervention, which includes two separate but complementary interventions that address MSW-specific behavioral, economic, interpersonal and structural-level barriers to accessing and adhering to PrEP: (1) peer-led, SBCM for PrEP linkage and uptake, and (2) a technology and counseling intervention to optimize PrEP adherence (once they have access to PrEP). This research, reviewed and funded by the National Institutes of Health, aims to test the “PrEPare for Work” intervention in a fully-powered, two-stage, parallel RCT to assess efficacy. The SPIRIT figure of this trial, including the schedule of screening, enrollment and follow up visits, is illustrated in Fig. 1.

### Participant recruitment and screening

Participant recruitment involves active and passive methods. Study staff will recruit and enroll 500 men over 42 months nationally. Enrollment is offered in person (when geographically feasible– e.g., in Greater Providence or Greater Los Angeles areas) and remotely. Study staff carry out active recruitment and enrollment at our two primary sites, the Brown University School of Public Health and UCLA Fielding School of Public Health. Each site plans to enroll approximately 250 participants. The research staff receive additional support with recruitment and retention from Project Weber/RENEW (Providence, RI), The Miriam Hospital (Providence, RI), Open Door Health (Providence, RI), and the UCLA Center for LGBTQ + Advocacy, Research & Health (Los Angeles, CA).

In addition to identifying participants through our active partner organizations, participants are recruited from a variety of sources: [1] members of our recruitment/outreach team actively recruit participants in the community via direct outreach at venues where we know sex workers solicit clients (such as bars, night/dance clubs, on the streets), [2] identifying additional partners with strong and trusting ties in the community, [3] via the internet, by posting study-related advertisements, [4] via direct engagement with online escorts who solicit sex for pay, and [5] via snowball techniques. Recruitment is currently ongoing.

Eligibility screening may be conducted in person during outreach activities, over the phone in response to posted advertisements, and via a live link embedded in online advertisements. We also have flyers and palm cards to advertise the study. If eligibility is conducted by phone, research staff briefly explain the study to interested volunteers and assess interest in being screened for eligibility. Once a participant is determined to be eligible post-screening, a baseline appointment is scheduled. If eligibility is administered via a live link, a brief introduction is provided prior to the volunteer being prompted to complete an online survey. Online eligibility screeners are subject to eligibility confirmation verbally (i.e., by phone, in person, or via video conference) by research staff before the informed consent process. See Table 1 for eligibility criteria.

**Table 1:** Eligibility Criteria for PrEPare for Work Study

Inclusion Criteria	Exclusion Criteria
• Age: 18 years or older • Assigned male sex at birth • Identifies as male at enrollment • Report having exchanged sex for money, drugs, a place to stay, or any items of value with another man within the past three months • Not currently on PrEP • Indicated for PrEP per CDC guidelines (including HIV uninfected by antibody test) • Owns a cell phone or is willing to use one as part of the study • Able to understand and speak English	• Unable to provide informed consent due to severe mental or physical illness, cognitive impairment, or substance intoxication at the time of interview (will use an adapted version of the Evaluation to Sign Consent Form^11^ to assess capacity) • Discovery of active suicidal ideation or serious mental illness (e.g., current psychosis or mania) at the time of interview (these patients will be referred immediately for treatment but may join the study when this is resolved) • Was randomized to the intervention condition of the pilot RCT

### Informed consent and enrollment

After confirming eligibility, trained study staff conduct informed consent in a private location using a consent document that describes the study rationale, procedures, risks, benefits, confidentiality, and rights and responsibilities. Study staff then ask potential participants questions to ensure comprehension. Individuals who consent to participate then e-sign the informed consent form.

### Randomization

Following baseline assessment administration and prior to beginning study intervention, randomization occurs. Study staff uses site-stratified computer-generated block randomization with alternating block numbers ranging from 4 to 8, so study staff cannot guess the study condition. Randomization is implemented electronically through REDCap.

### Timing of assessments

**Fig. 2:**
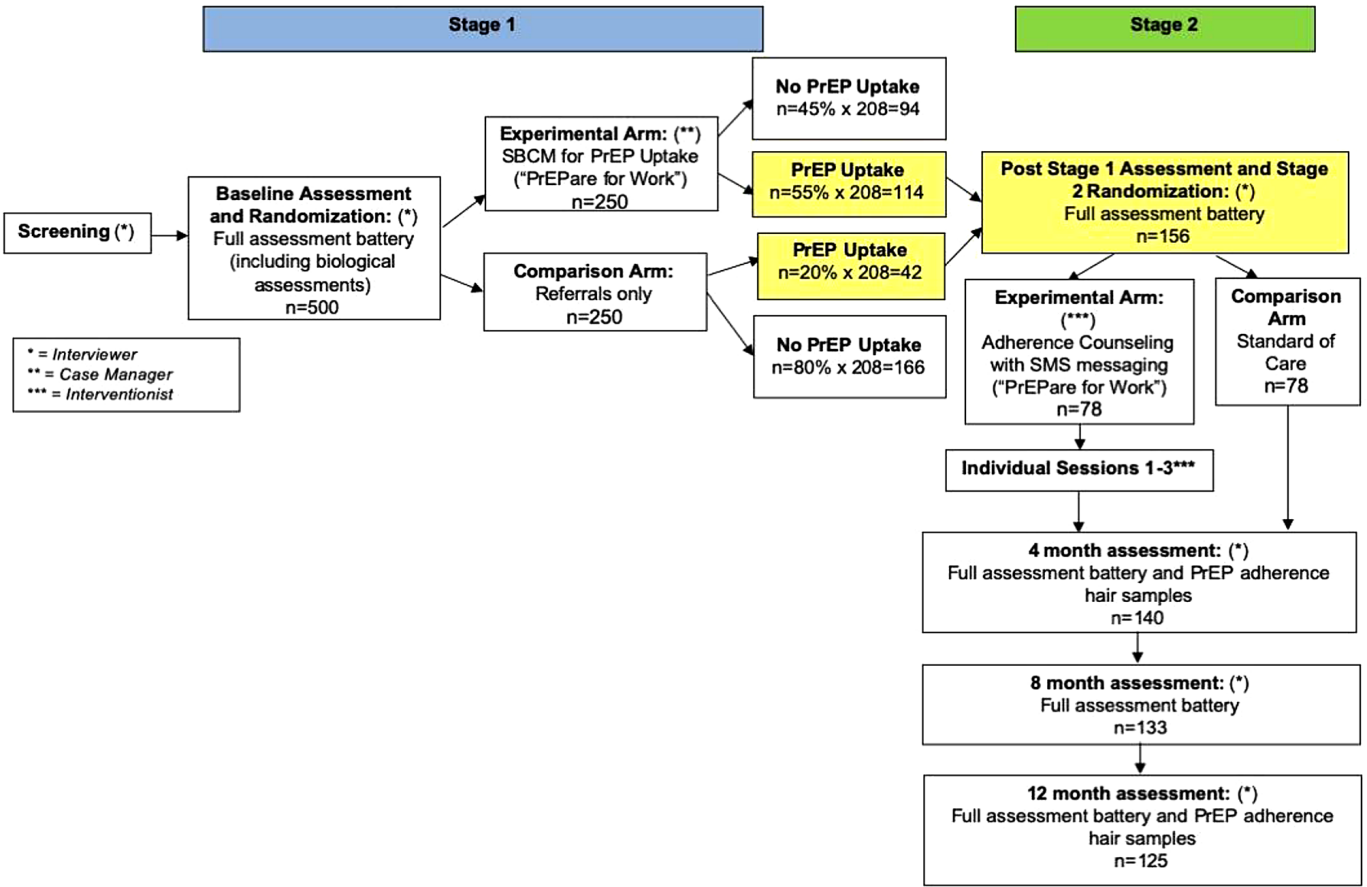
Flow chart of study design with schedule of activities and estimated sample sizes for PrEPare for Work study

See Fig. 2 for details. Stage 1 has up to 3 major assessment points: Stage 1 Baseline (pre-randomization) and Post Stage 1 Assessments (at 1- and 2-months or at PrEP initiation, whichever occurs first). Participants who initiate PrEP (confirmed by a verified PrEP prescription in the participant’s name) complete the Stage 2 Baseline instead of the Stage 1 follow-up assessment. For participants who do not initiate PrEP, the 2-month follow-up is the final research assessment.

Stage 2 has four major assessment points: Stage 2 Baseline (pre-randomization), acute follow-up (4 months), and longer-term follow-ups (8 and 12 months). At each Stage 2 assessment, PrEP adherence is assessed via self-report; at 4-, 8-, and 12-month visits, a hair sample is collected to measure PrEP drug level in hair (long-term adherence). At the Stage 2 Baseline visit, a medical release is completed to obtain specific clinical indicators and associated HIV/STI testing results throughout the study period. At the 4- and 12-month visits, participants complete a self-collected HIV test and a urogenital (urine) test for chlamydia and gonorrhea. At all major assessments, participants complete a self-report battery, which includes secondary outcomes, a comprehensive psychosocial assessment of moderating factors, and potential mediators of the intervention.

Assessments are not blinded; however, only research staff who do not deliver intervention content administer follow-up assessments to reduce social desirability bias. Moreover, study staff use standardized survey instruments and are instructed on how to avoid bias in survey administration. Participants receive compensation for all study visits and may complete portions of study assessments remotely via a HIPAA-secured Zoom platform if desired. Between major assessments, check-ins (by phone or in-person) to support retention are incentivized.

### “PrEPare for Work” strength-based case management intervention condition (stage 1)

Following Stage 1 randomization, participants randomized into the intervention arm are provided a study case manager (CM) to motivate, support, facilitate, and assist in linkage to established PrEP clinics and to facilitate initiation and obtainment of PrEP medications. CM staff are lay individuals (i.e., no certifications or licensing requirements) who have experience working with MSWs or other high-risk populations and have received extensive training to support their work (e.g., harm reduction, motivational interviewing, professional boundaries).

The “PrEPare for Work” SBCM intervention consists of two structured (incentivized) case management sessions: one scheduled following Stage 1 randomization and one scheduled according to individual participant needs and before the 2-month follow-up visit. In addition to these visits, the CM is available to provide support and services throughout the intervention (i.e., two months). The goal of the facilitated SBCM services is to reduce initial barriers to taking PrEP by providing information (e.g., what PrEP is and its function), motivation (e.g., how one may benefit from PrEP uptake) and support for identifying and problem-solving relevant barriers (e.g., finding and contacting a provider, getting transportation to a clinic/pharmacy).

### “PrEPare for Work” adherence intervention condition (stage 2)

The “PrEPare for Work” intervention consists of adherence training and counseling, as well as daily text messages. The adherence training and counseling consists of three one-on-one sessions, lasting approximately 45–60 min, with a clinical social worker (or someone with similar training). In these sessions, the counselor and participant discuss general PrEP information and the rationale for PrEP adherence. They discuss the participant’s sexual behavior patterns (particularly in the context of sex work) HIV risk limits, and barriers and facilitators of staying within these limits. Additionally, they discuss motivations for PrEP use, assess potential barriers to optimal adherence (e.g., stigma, sex work clients, substance use, mood, housing instability) and problem-solve these barriers. This is done in a client-centered, nonjudgmental way to facilitate honest discussion.

Daily text messages are deployed to those randomized to the “PrEPare for Work” intervention condition. Messages are sent according to their medication schedule/time they take PrEP each day for four months. Text message reminders serve not only as indications to take PrEP as prescribed but also as cues for behavioral skills gained during in-person adherence counseling sessions. Study staff program text messaging software to send daily reminders to relevant participants immediately following randomization. We encourage participants to delete text messages after taking their medication and use confidential messages that do not mention PrEP-specific medications or the present study. Participants choose from one of two sets of text messages, which may be changed as requested throughout the study period. Examples of personalization include: “Don’t forget!” “Stick with it!” “Your health comes 1st.” Participants can choose to opt out of receiving text messages. Study staff periodically check in with participants who opt out of receiving text messages about their ability to opt back in at any time during study participation.

### Standard of care control condition

For Stage 1 (PrEP linkage/uptake), the SOC condition consists of the provision of resources including information about oral and injectable PrEP (what they are, how they work, their efficacy), how to pay for PrEP with or without insurance, and a list of local resources for mental and sexual health, primary care, sexual assault, domestic violence, substance use, and HIV/STI care and prevention. Irrespective of the study condition, if they attend a PrEP appointment, participants can be prescribed PrEP at the discretion and following the protocol of the prescribing clinician.

### Measures

At all major assessment visits, participants complete interviewer-administered assessments regarding socio-demographics, HIV and PrEP knowledge, perceived HIV risk, PrEP interest and self-efficacy, substance use, sexual behaviors, physical and psychosocial health conditions, and health care utilization. Medical record review is conducted throughout the second stage of the study. Biological adherence measures (i.e., hair samples) are collected at 4-, 8- and 12-month follow-up visits only.

#### Co-primary outcome measures

PrEP uptake is measured via confirmation of medical and pharmacy records. We obtain a release of medical information from participants at the Stage 2 baseline. Study staff follow up with PrEP providers (to verify clinical PrEP eligibility and visit attendance) and pharmacies (to determine whether prescriptions for TDF/FTC or TAF/FTC were filled and dates when filled to assess time to initiation). Study staff also request at each follow-up assessment that participants show their pill bottles to confirm that the prescription was picked up.

For those who initiate PrEP, longer-term (past month) adherence is assessed at 4-, 8, and 12-month visits by collecting hair samples to test for detectable levels of TDF/FTC or TAF/FTC. Samples are ~ 50–100, 1-2 mm-long hair strands cut with scissors. As in our other studies, samples are stored and shipped to the University of California San Francisco Hair Analysis Lab (HAL) for analysis. Although this method was highly feasible and acceptable in our pilot (> 95% provided samples) [17], participants refusing to provide hair (or those without sufficient hair) can continue to participate. Self-reported adherence supplements biological monitoring at all major assessments. In line with our prior trials and available evidence regarding the validity and reliability of self-reported adherence assessments, participants are asked to rate their adherence frequency (e.g., “all the time”) and report missed doses [23]. Additionally, retention in PrEP care is assessed based on the number of PrEP visits attended over 12 months of follow-up (after initial appointment, 2 + appointments will be considered optimal retention).

#### Secondary outcome

PrEP persistence is assessed using pharmacy and medical records to confirm prescription refill maintenance. As per Coy et al., PrEP persistence is defined as having ≥ 16 days of PrEP medication filled per 30-day period for at least three-quarters of those months from initiation to study completion (~ 9 months) [24].

#### Conceptual mediators

Guided by our formative work and our conceptual model [14, 15, 17, 21, 22, 25, 26], variables hypothesized to be mediators of the intervention effect, including HIV and PrEP adherence information, PrEP motivation, and behavioral skills, are measured at each major assessment. Assessments are based on adaptations of validated scales [27, 28] and were used in our pilot study [17].

#### Hypothesized moderators

According to our model, the following moderators and descriptive measures are assessed: Socio-demographics (age, sexual identity, race/ethnicity), psychosocial (depressive and anxiety symptoms, trauma and abuse), substance use, and sex work disclosure.

### Statistical analyses

#### Aim 1

The primary analysis for Stage 1 will compare PrEP initiation (operationalized as having been prescribed PrEP medication) by two months post-Stage 1 randomization between the study arms. For Stage 2, the primary analysis will compare adherence (operationalized by detectable PrEP drug level in hair) at the 4-, 8-, and 12-month visits between study arms. Moreover, group differences in the number of PrEP clinic appointments kept and changes in self-reported adherence and sexual behavior adjustment will also be compared. All analyses will use two-tailed significance tests, with significance at alpha = 0.05. For each analysis, we will use generalized linear models (GLMs) with properly chosen link functions to analyze longitudinal data. The GLMs will be estimated using generalized estimating equations with robust standard error estimates (GEE), which provides an extension of regression analysis to the case of correlated or repeated observations and allows for the inclusion of both categorical and count dependent variables, as well as for appropriate modeling of covariance structures when observations are correlated across time [29, 30]. We will follow an intent-to-treat model, analyzing participants according to the study arm to which they were assigned, regardless of fidelity to the assigned group. Participants in Stage 2 who miss two or more sessions will be categorized as “non-completers” and analyzed secondarily in sensitivity analyses (dose-response relationship).

#### Aim 2

For mediation analyses, path analysis will be conducted using structural equation modeling (SEM) to determine whether the effect of the intervention on uptake and/or adherence was through the hypothesized mediators (e.g., increased PrEP knowledge, increased problem-solving skills to take PrEP, increased self-efficacy for PrEP adherence). SEM allows for the simultaneous estimation of total, direct, mediated, and indirect effects of a causal variable (i.e., the intervention) on the outcome (i.e., adherence) through a set of mediator variables [31]. SEM can handle outcomes and mediators with a variety of distributions (including Gaussian, Poisson, and Binomial). Inferences for indirect effects will be estimated using bootstrapped confidence intervals [32]. For effect modification (moderation) analyses, we will add interaction terms one by one for the intervention condition and the potential moderators (e.g., race, substance use at baseline, psychosocial factors; see Measures). Significant or large interaction terms suggest that intervention effects differ by subgroups of the moderators.

#### Aim 3

For the cost-effectiveness analysis, the costs of providing this intervention relative to the SOC arm will be estimated based on detailed records of resources required to implement the intervention from an organizational perspective, including the average personnel cost per participant for each intervention arm. The lifetime treatment cost of an HIV infection will be used as a conservative threshold value for the cost of averting one infection. The intervention will then be deemed cost-saving if it costs less than this threshold.

### Sample size calculations

The primary power analysis is based on the acute PrEP adherence outcome (differences between the intervention and control conditions from our “PrEPare for Work” pilot RCT), which demonstrated a meaningful but non-significant difference in PrEP adherence between the intervention and control groups from baseline to follow-up (effect size: 0.54) [17]. Therefore, the present study is powered to detect an effect size of d = 0.54 using a two-sided alpha = 0.05. Group sizes of 63 completers per arm (experimental intervention and the standard of care comparison), which assume 20% attrition in Stage 2, result in greater than 80% power in adherence at four months in a pair-wise comparison. Assuming similar rates of uptake and attrition to the pilot study, 250 individuals per arm are required for randomization.

### Data management, safety and monitoring

All survey data is inputted directly into REDCap, a HIPAA-compliant, comprehensive data management system. Hard and soft-copy participant data is identified by an ID number only, and a link between names and ID numbers is kept separately in a password-protected file. Likewise, name-based files are stored separately from survey data. Soft copy data is stored on study-specific secure and password-protected network drive folders, accessible only to study staff. Hard copy data is stored in locked cabinets within restricted and secure areas at study sites.

All study staff are trained in confidentiality and have signed confidentiality agreements. Study staff have been trained in ethical human subject research practices to minimize participant risk. The investigators report unanticipated problems, safety monitors’ reports, and adverse events to the University of California, Los Angeles IRB, per IRB policies. Any protocol modifications will be reported to and approved by the UCLA IRB.

Given that this is a behavioral intervention with minimal risk, the study has no stopping rules, and interim analyses are not conducted. An independent Data Safety Monitoring Board has been assembled and reviews study progress, including safety concerns and adverse events, twice annually. All reports are shared with the IRB and funder at least annually.

### Dissemination plan

In addition to reporting on clinicaltrials.gov, findings from this study will be disseminated via peer-reviewed publications and conference abstracts/presentations. Moreover, presentations at community organizations, including our partner SSP sites, and government entities (e.g., local public health departments, CDC) will be conducted. We will also create easy-to-read infographics to share via social media and our research group website(s).

## Discussion

The “PrEPare for Work” intervention is the first theory-based PrEP uptake and adherence intervention for MSWs, who are among the most marginalized, at-risk and understudied populations in the U.S. “PrEPare for Work” was informed, developed, and refined through formative community-based research that involved MSWs at each stage to ensure study implementation and design, as well as intervention content addresses and honors their lived contextual realities and that those who are most in need benefit from this transformative biomedical HIV prevention modality. For example, as many MSWs are not publicly open about their involvement in sex work or are afraid of legal ramifications, our recruitment materials use less explicit language to highlight paid research opportunities about men and sexual health.

The integration of text messaging technology to support adherence, which we found to be highly acceptable and feasible in the pilot RCT of this intervention [17], has been used successfully with other populations [33, 34] but has not been used previously with MSWs — given the broad use of mobile phones to connect with both clients and personal networks, this component has unique promise. Furthermore, the design of “PrEPare for Work” allows us to efficiently test both the PrEP initiation and PrEP adherence components of the intervention, which aligns with real-world clinical settings — firstly, PrEP is provided only within usual clinical care; and secondly, if a person is not ready nor feeling empowered to initiate PrEP, an adherence intervention is not responsive nor an appropriate use of resources. Thus, this study is testing an intervention within a real-world context and hence may provide evidence of a sustainable and scalable intervention, which could be adapted for other sex worker populations in the US and potentially globally and among other groups with shared risk factors. Ultimately, if efficacious, this intervention could be integrated within existing healthcare systems and community harm reduction organizations.

There are potential limitations to the proposed project. Firstly, the RCT may not be powered to determine intervention efficacy for our secondary outcome of PrEP persistence; however, findings may still help inform subsequent research. Secondly, the formative research and pilot study was comprised of primarily street-based sex workers in the U.S. Northeast [11, 17, 25, 35]. Given that the present study aims to broaden its sample across geography and sex work type, its content may need to be slightly adapted to ensure relevance across diverse potential participants. Importantly, our mediation and moderation analyses will provide more insight into those mechanisms of action and subgroup differences, which can inform future broadening in scope.

In sum, based on formative work and community collaboration, we developed the theory-informed, multi-component “PrEPare for Work” intervention targeting PrEP knowledge, motivation, self-efficacy, behavioral skills, and structural barriers to access among MSWs at risk of HIV acquisition. Importantly, this RCT plans to enroll MSWs in diverse geographic areas and with diverse sex work and lived experiences, providing insight into the generalizability of this intervention. If efficacious, findings could inform the dissemination of “PrEPare for Work” to community-based and clinical settings throughout the U.S.

## Data Availability

Reasonable requests to access study data will be considered by the corresponding authors(s), and upon completion of planned study outcome analyses, raw data will be freely available to any scientist wishing to use them for non-commercial purposes, without breaching patient confidentiality, per journal guidelines.
